# Exclusion of CLIC5 as a Candidate Gene and Identification of NEFM as a Possible Novel Gene Correlated With Autosomal Recessive Pure Cerebellar Ataxia in a Highly Consanguineous Family

**DOI:** 10.1002/mgg3.70199

**Published:** 2026-03-30

**Authors:** Paolo Enrico Maltese, Gabriele Bonetti, Elena Manara, Benedetta Tanzi, Andrea Bernini, Mark A. Berryman, Soichi Tanda, Corinne Nielsen, Silvia Casagrande, Amanda Ferrero, Salvatore Stano, Riccardo Zuccarino, Andrea Barp, Pietro Chiurazzi, Matteo Bertelli

**Affiliations:** ^1^ MAGI'S LAB Rovereto Italy; ^2^ Department of Pharmaceutical Sciences University of Perugia Perugia Italy; ^3^ Department of Biotechnology, Chemistry, and Pharmacy University of Siena Siena Italy; ^4^ Department of Biological Sciences Ohio University Athens Ohio USA; ^5^ Neurology Unit, Rovereto Hospital Trento Italy; ^6^ Fondazione Serena Centro Clinico NeMO Trento, APSS Trento Italy; ^7^ Medicina Genomica, Dipartimento di Scienze Della Vita e sanità Pubblica Università Cattolica del Sacro Cuore Rome Italy; ^8^ UOC Genetica Medica, Fondazione Policlinico Universitario ‘A. Gemelli’ IRCCS Rome Italy; ^9^ MAGI Euregio Bolzano Italy; ^10^ MAGISNAT Peachtree Corners GA USA

**Keywords:** cerebellar ataxia, CLIC5, NEFM, WES

## Abstract

**Background:**

Pure cerebellar ataxia is a neurological disorder characterised by isolated cerebellar dysfunction, arising from either developmental anomalies or progressive degenerative processes. Precise genetic diagnosis remains challenging.

**Methods:**

The aim of this study was to use a whole‐exome sequencing approach to study a large, highly consanguineous Italian family in order to identify a new gene correlated with pure cerebellar ataxia.

**Results:**

Sequencing excluded the presence of mutations in known‐related genes but revealed a homozygous missense variant in *CLIC5*; however in vivo analysis of a *CLIC5* KO mouse model showed vestibular dysfunction without cerebellar involvement, suggesting that *CLIC5* is not directly involved in pure cerebellar ataxia onset. Further analysis identified two compound heterozygous variants in *NEFM*, and *in silico* analysis showed that they dysregulate NEFM phosphorylation. Phosphorylation of neurofilaments and subsequent formation of aggregates has already been linked to conditions such as ageffing and neurodegeneration. Moreover, in vivo studies on mice transgenic for human *NEFM* have correlated NEFM phosphorylation and aggregation with neurodegeneration. Finally, neurofilaments have been proposed to be correlated to ataxia and autoimmune cerebellar ataxia.

**Conclusion:**

We therefore propose *NEFM* as a possible new candidate gene for hereditary cerebellar ataxia. These findings could be useful for advancing the genetic diagnosis of hereditary pure cerebellar ataxia, possibly enabling the screening of healthy carriers.

## Graphical abstract

Whole‐exome sequencing candidate *CLIC5* and *NEFM* for cerebellar ataxia (CA) onset. In vivo studies exclude *CLIC5*, while *in silico* analysis and literature review support *NEFM* as a candidate gene for CA. These findings could be useful for advancing the genetic diagnosis of hereditary pure CA. Created in https://BioRender.com.

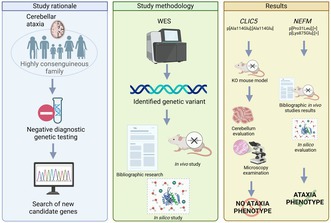

## 1 Introduction

Pure cerebellar ataxia (CA) is a neurological disorder which affects the cerebellum and is characterised by movement incoordination (Kerber et al. 2005). Apart from influencing motor function, CA influences cognition and mood, emphasising the multifaceted role of the cerebellum in human physiology. The cerebellum controls limb and eye movements, including balance and walking. Symptoms of CA encompass visual and speech ataxia, dysmetria, dyscoordination, and ataxia (Fogel 2012; J. F. Marsden 2018; Marsden and Harris 2011). Considering the wide variety of symptoms and possible early onset, the diagnosis of CA remains challenging. It calls for comprehensive clinical examination and medical history. Typical signs, including gait disturbances, dysmetria, and impaired coordination, are evaluated. Advanced diagnostic tools, such as neuroimageing (MRI and CT scans) and laboratory analysis, are useful for identifying structural abnormalities or underlying conditions contributing to cerebellar dysfunction (Marsden 2018; Radmard et al. 2023).

The complex spectrum of CA symptoms and etiologies makes the disorder difficult to treat. While there is currently no cure for most hereditary forms of CA, a comprehensive management may alleviate symptoms, enhance functional capabilities and improve overall quality of life (Jones et al. 2014; J. Marsden and Harris 2011). Three main approaches are used to manage CA: (i) physical and occupational therapy, (ii) assistive devices, and (iii) pharmacological treatments. (i) Physical and occupational therapy focuses on improving balance, coordination and mobility, with specific exercises targeting gait, posture and fine motor skills. (ii) Assistive devices comprise walking aids such as canes and mobility scooters, as well as adaptive tools for daily activities, such as utensils with larger grips or devices with voice‐activated controls. (iii) While there is no specific medication to cure cerebellar ataxia, certain drugs such as aminopyridines and anti‐seizure drugs may be prescribed to alleviate symptoms or manage associated conditions (Jones et al. 2014; Marsden and Harris 2011). The complexity of CA demands a holistic approach that begins with precise diagnosis and extends to customized therapeutic strategies.

After clinical examination, genetic testing is important for an accurate diagnosis when a genetic basis is suspected. Hereditary forms of CA are clinically and genetically heterogeneous. They are classified according to mode of inheritance, which may be autosomal dominant, autosomal recessive or X–linked; there are also mitochondrial forms. Friedreich's ataxia is the most common form of autosomal recessive cerebellar ataxia (Palau and Espinós 2006). Advances in molecular techniques, particularly next‐generation sequencing (NGS), have facilitated rapid cost‐effective detection of causative variants (Krygier and Mazurkiewicz‐Bełdzińska 2021; Sailer and Houlden 2012). Despite extensive research, a definitive diagnosis remains elusive for many affected individuals. In a recent study of more than 250 patients analysed for rare coding variants, copy number variations and repeat expansions, a total clinical detection rate of around 50% was obtained, meaning that new approaches are needed to define all the causes of CA (Ngo et al. 2020).

Gene discovery in autosomal‐recessive CAs, starting with single‐gene discovery to a high‐throughput search, has enabled the identification of new disease mechanisms. Early molecular work in Friedreich's ataxia uncovered the inheritance of expanded CAG trinucleotide repeat in frataxin, thus proposing mitochondrial dysfunction as a main pathogenic mechanism (Klockgether 2000; Klockgether and Evert 1998). Subsequent studies extended the CA‐correlated genes to include *ATM*, *SACS*, *APTX*, *SETX*, *POLG*, *TTPA*, *PNPLA6*, *ANO10* and other loci (Vermeer et al. 2010; Wiethoff et al. 2017; Storey 2014). The introduction of NGS, and especially whole‐exome sequencing (WES) and trio‐based approaches, emerged as a powerful diagnostic tool that can significantly increase the overall diagnostic yield for genetic ataxia. Indeed, these techniques identified new variants in genes involved in several molecular pathways, such as DNA repair, ion homoeostasis and membrane trafficking, increasing the overall number of genes correlated to autosomal‐recessive cerebellar ataxias and revealing novel mutational mechanisms (Coarelli et al. 2023; Coutelier et al. 2015). The identification of new diagnostic and therapeutic biomarkers could help in the diagnosis and management of patients with CA. The aim of the present study was to use a WES approach to identify a new gene possibly correlated with an autosomal recessive form of CA.

## 2 Methods

### 2.1 Editorial Policies and Ethical Considerations

The study complied with the Declaration of Helsinki and was approved by the local ethics committee (Comitato Etico dell'Azienda Sanitaria dell'Alto Adige, Protocol number 0111181‐BZ).

### 2.2 Patient Recruitment and Sample Preparation

**FIGURE 1:**
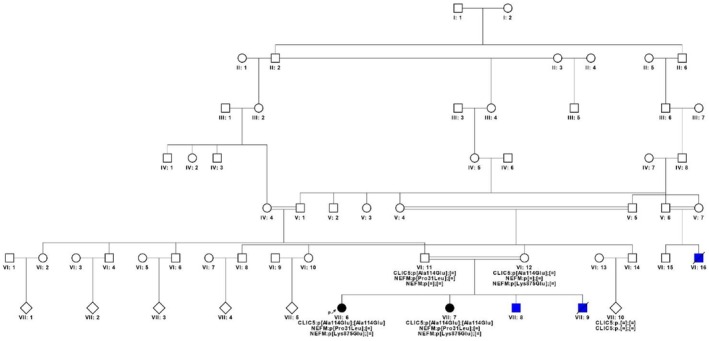
Pedigree of seven generations of the family. CA. Dystrophic bullous epidermolysis.

Five members of a family already studied and reported (Colombi et al. 1991, 1992) were recruited. The family lives in an isolated region of northern Italy. Over seven generations, two common ancestors and four marriages between consanguineous individuals occurred. For the present study, we focused on the most recent branch of the family (Figure 1). All patients received pre‐test genetic counselling and provided written informed consent in compliance with the Declaration of Helsinki.

### 2.3 Genetic and Data Analysis

Approximately 5 mL of peripheral blood or saliva from the probands was used for DNA extraction with a commercial kit (Blood DNA kit E.N.Z.A., Omega Bio‐tek Inc., Doraville, GA, USA or Exgene Clinic SV Mini, GeneAll Biotechnology, Seoul, South Korea). The family tree was built with HaploPainter software (HaploPainter. Available Online: https://Haplopainter.Sourceforge.Net/Download.Html (Accessed on 29 December 2023)). Pathogenicity predictions for missense variants were obtained using three widely used computational tools: MutationTaster (https://www.mutationtaster.org/), SIFT, and PolyPhen‐2, both accessed via the Variant Effect Predictor (VEP) tool on Ensembl (https://www.ensembl.org/Tools/VEP). Each tool assigns qualitative predictions based on evolutionary conservation, protein structure, and biochemical impact of amino acid substitutions.

### 2.4 Whole‐Exome Sequencing

The analysis, performed by IntegraGen SA (Evry, France), was conducted by sequencing the exons and intron–exon junctions of all known genes on an Illumina NovaSeq sequencer using the Twist Human Core Exome Enrichment System (Twist Bioscience) + IntegraGen Custom V2 protocol. Under the hypothesis of an autosomal‐recessive disorder, the analysis first focused on homozygous variants in the patients and heterozygous variants in the parents and then on compound heterozygous variants in daughters and simple heterozygous variants in the parents. Next, homozygous or compound heterozygous variants present in control family members, in the laboratory's internal controls analysed by the same protocol, and in more than 1% of subjects in two population databases were eliminated. Finally, variants not expected to induce an amino acid change or other serious protein sequence alterations were eliminated.

### 2.5 Animal Model

*Clic5* mutant mice were derived from the C57BL/6J‐*Clic5* ^*jbg‐2J*^/KjnJ strain (JAX stock #010936). Since this stock carries an *ahl* mutation, *Cdh23* ^*c.753A*^, we replaced the *Cdh23* ^*c.753A*^ allele with the *Cdh23* ^*c.753G*^ allele in the B6N(Cg)‐Cdh23^tm2.1Kjn^/Kjn strain (JAX stock #18399) to avoid any potential effects of the *ahl Cdh23* ^*c.753A*^ allele. *Clic5* ^*jbg‐2J*^ homozygotes and heterozygotes were selected either by observing typical behavioural phenotypes (*MGI J:276827*, n.d.) or by PCR‐based genotyping using three primers, wild‐type forward (5′ GTCATCCCATGGGTAATGCT 3′), mutant forward (5′ GATTTGCCTGCATGTGTGTC 3′), and common reverse (5′ AGGCCTTTTCTCACCATCCT 3′) primers, in a single PCR reaction. Expected amplicons were a 306 bp band for the wild‐type allele and/or a 192 bp band for the *Clic5* ^*jbg‐2J*^ allele. For *Cdh23* genotyping, the forward (5′ CTTAAGCTCGGCCAAACATC 3′) and the reverse (5′ CACAACAGGAAGAAAGCAAGC 3′) primers were used as previously described (Johnson et al. 2017). Male mice between 8 to 9 months old were used for experiments. All experimental procedures were performed at Ohio University and approved by the Institutional Animal Care and Use Committee, Protocols # 14‐H‐028 and # 16‐H‐024. All methods were performed in strict accordance with NIH guidelines.

### 2.6 Measurement of Cerebellar Area

At tissue harvest, mice were transcardially perfused with 4% paraformaldehyde. Brain tissue was removed from the skull, immersed in 4% paraformaldehyde overnight at 4°C, and processed for paraffin embedding (Histoplast PE, Fisher Scientific). Sagittal brain tissue sections (8 μm) were collected and stained with haematoxylin and eosin, following standard technique. Images of whole‐mount and sagittal sections were acquired using a Nikon SMZ745 microscope and Nikon Elements software. Cerebellar areas and lengths were outlined and measured using FIJI software (National Institutes of Health, USA). The investigator was blinded to tissue genotype. The cerebellar peduncles were not included in the area measurements.

### 2.7 Data Analysis and Statistics

Quantitative data were plotted and analysed using GraphPad Prism software. Data was analysed by the Mann–Whitney and Student's *t*‐tests with Welch's correction. Statistical significance was set at α < 0.05.

## 3 Results

We studied a highly consanguineous family from Trento province in northern Italy (Figure 1), where two patients (VII:6 and VII:7), daughters of first cousins (VI:11 and VI:12), presented with pure CA after puberty. This family had already been studied because three other individuals (VI:16, VII:8 and VII:9) had autosomal recessive dystrophic bullous epidermolysis (Colombi et al. 1991, 1992). Clinical examination showed that the two sisters with CA had cerebellar signs (dysmetria of the upper and lower limbs, speech ataxia, wide‐based stance and gait with oscillations in many directions) that worsened over the years, without any pyramidal or sensitivity alterations. Clinical examination indicated that these patients had pure CA with autosomal recessive inheritance.

### 3.1 Genetic Results

Based on the clinical presentation characterised by ataxia, dysarthria and cerebellar hypoplasia, WES analysis was conducted first analysing all the genes already known to cause familial CA, but no pathogenic variants were found in genes that cause known forms of ataxia. We therefore focused our attention on candidate genes for ataxia and, given the consanguinity of the parents and the likely autosomal‐recessive mode of transmission, we looked for homozygous variants detected in the probands (Table S1). After selecting genetic variants with MAF < 0.01, considering tissue expression of the genes and performing a search of the literature for possible correlations between the detected genes and ataxia, *CLIC5* was selected as the most likely candidate for CA in this family. However, considering compound heterozygous transmission, we also selected *NEFM* as a candidate gene. The variants identified in *CLIC5* and *NEFM* are reported in Table 1. The selection of the variants began prior to the creation of the Genome Aggregation Database (gnomAD; http://gnomad.broadinstitute.org/). Therefore, in Table 1, we report data from the most recent gnomAD release (v4.1.0) and from the earliest available version (v2.1.1), which corresponds approximately to the period when the study was initiated. *In silico* pathogenicity prediction tools have been used to evaluate the variants. Results are reported in Table S2. No mutations of either gene have hitherto been found in patients with ataxia.

**TABLE 1:** List of genetic variants that co‐segregate with the phenotype and are compatible with autosomal‐recessive or compound‐heterozygous transmission.

Gene	Genetic variant	Zygosity	Amino acid variant	gnomAD v4.1 frequency	Homozygotes in gnomAD v4.1	gnomAD v2.1.1 frequency	Homozygotes in gnomAD v2.1.1
*CLIC5*	NM_016929:c.341C > A	Hom	p.(Ala114Glu)	0.002836 (4577/1613918)	8	0.002055 (581/282714)	3
*NEFM*	NM_005382.2:c.92C > T	Het	p.(Pro31Leu)	0.000002057 (3/1458368)	0	NA	NA
*NEFM*	NM_005382.2:c.2623A > G	Het	p.(Lys875Glu)	0.000003098 (5/1614122)	0	0.00001194 (3/251170)	0

*Note:* A homozygous variant in *CLIC5* and two compound‐heterozygous variants in *NEFM* were identified.

### 3.2 *CLIC5* – Measurement of Cerebellar Area and Histological Examination

**FIGURE 2:**
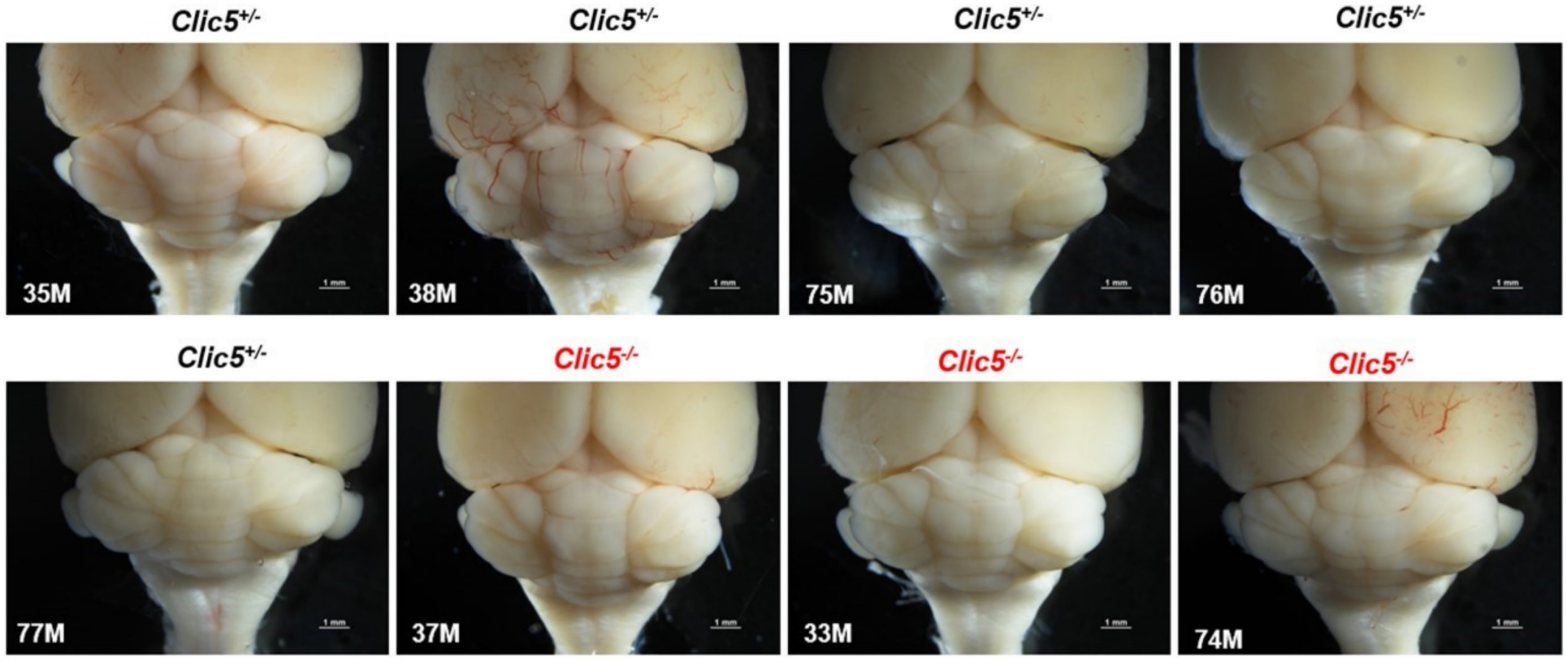
Whole cerebellum of heterozygous and homozygous *Clic5* mutant mice, dorsal view. Scale bars = 1 mm.

**FIGURE 3:**
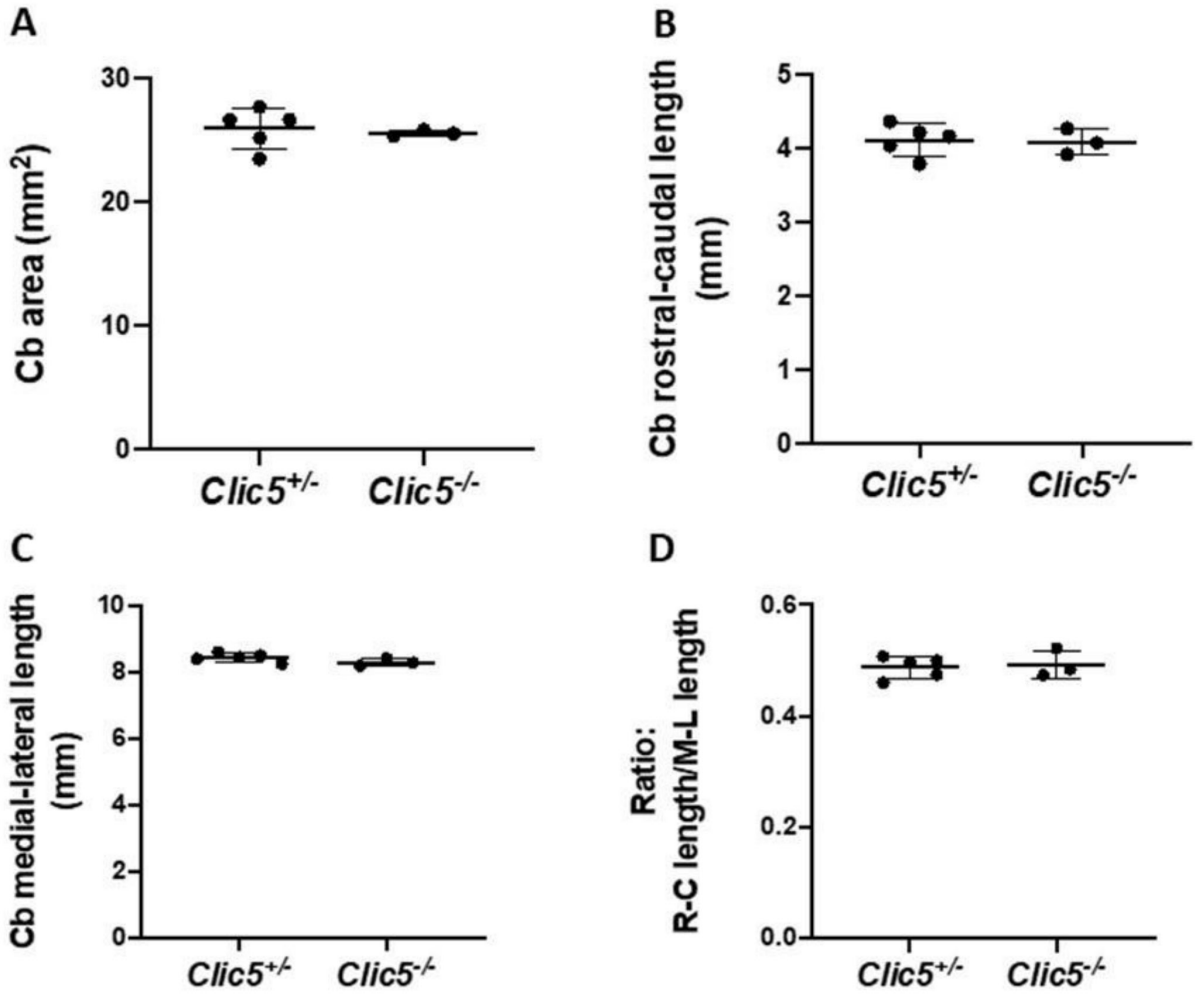
Whole‐mount cerebellar (Cb) morphometrics from dorsal view in heterozygous and homozygous *Clic5* mutant mice. (A) Cerebellar areas. (B) Cerebellar rostral–caudal (R–C) lengths. (C) Cerebellar medial–lateral (M–L) lengths. (D) Cerebellar R–C/M–L lengths.

**FIGURE 4:**
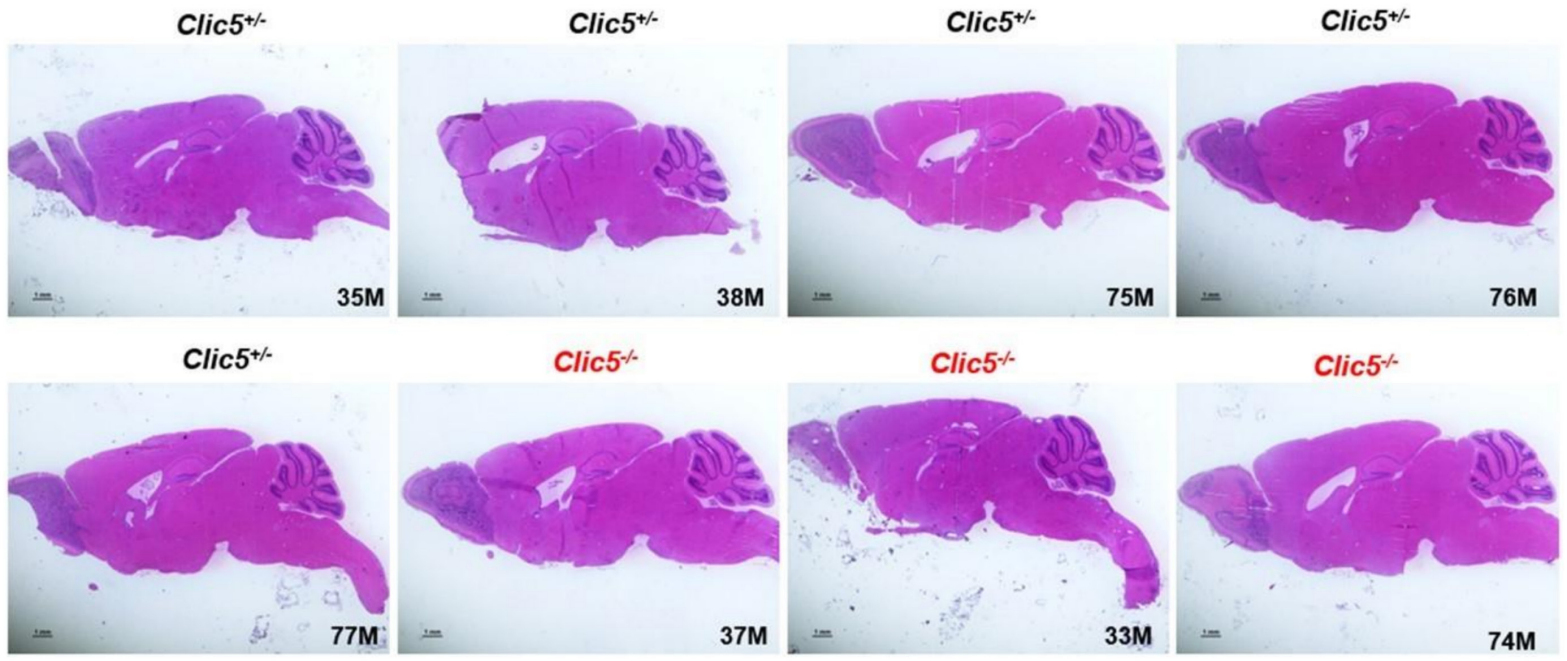
Histological sections of heterozygous and homozygous *Clic5* mutant mouse brains, midsagittal view. Scale bars = 1 mm.

**FIGURE 5:**
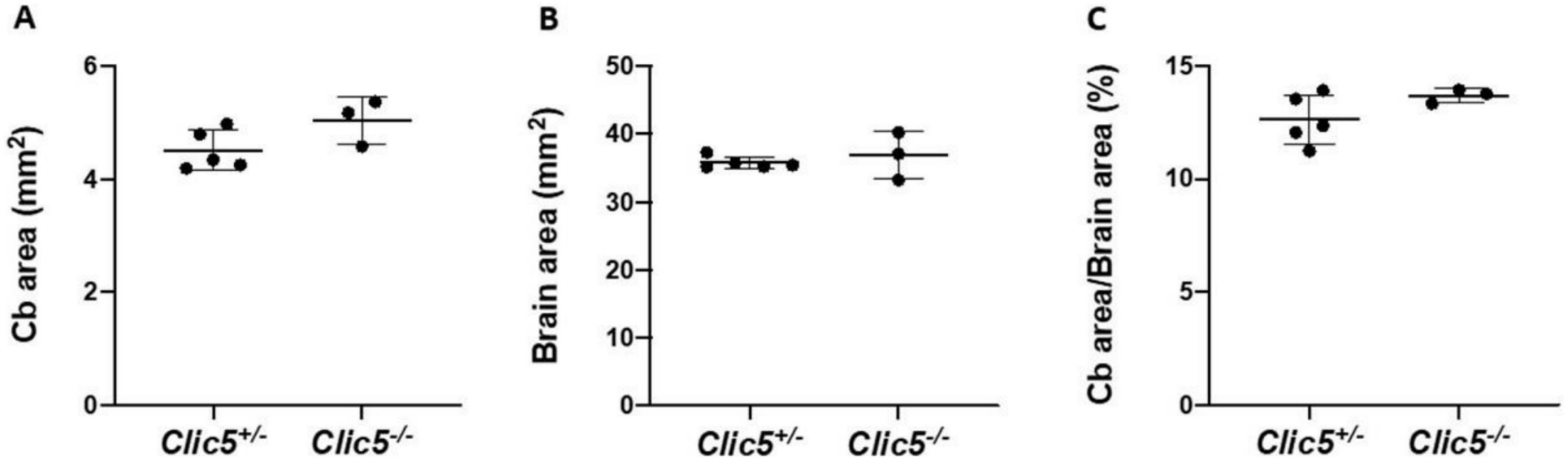
Cerebellar (Cb) morphometrics in sagittal sections from heterozygous and homozygous *Clic5* mutant mice. (A) Cerebellar (Cb) areas. (B) Brain areas. (C) Cerebellar area/Brain areas expressed as percentage.

**FIGURE 6:**
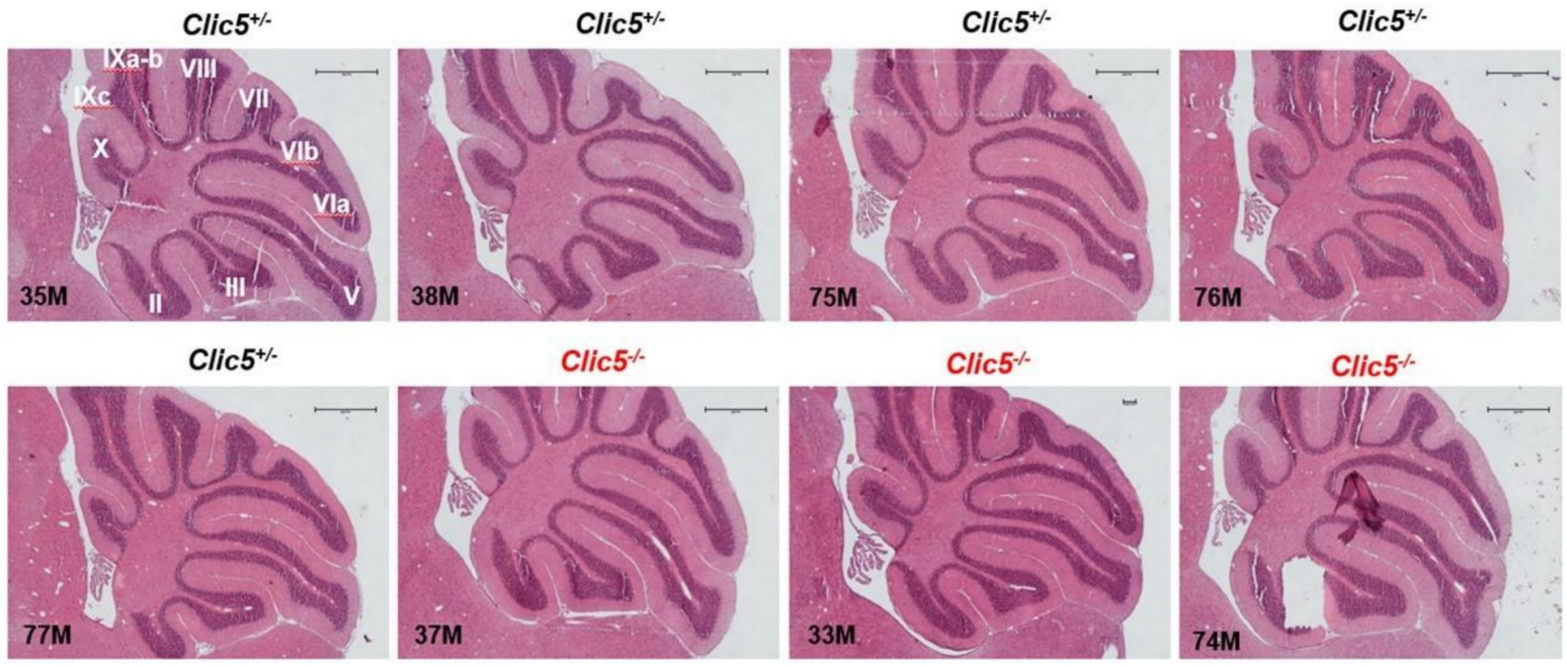
Histological sections showing cerebellar foliation patterns in heterozygous and homozygous *Clic5* mutant mice, midsagittal view. Foliation is indicated as vermis lobules II–X. Scale bars = 500 μm.

**FIGURE 7:**
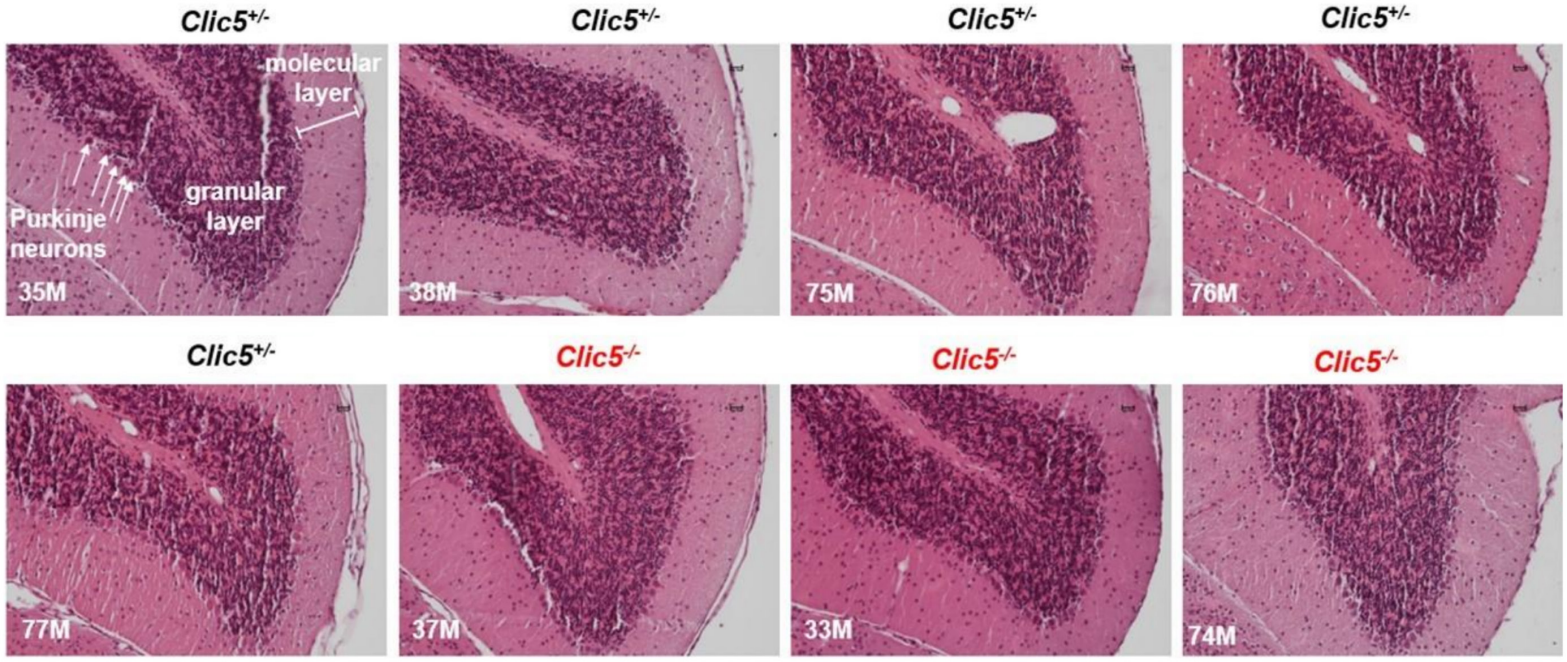
Histological sections showing cerebellar lamination in heterozygous and homozygous *Clic5* mutant mice, midsagittal view. Molecular, granular, and Purkinje neuronal layers were present in *Clic5* ^*+/−*^ *and Clic5* ^*−/−*^ mice. Scale bars = 20 μm.

In the gnomAD database, the p.(Ala114Glu) variant occurs in eight homozygous subjects and has a frequency of 4577 out of 1,613,918 alleles. Homozygous variants of *CLIC5* have been correlated with hearing problems and balance dysfunction in two distinct families (Seco et al. 2015; Wonkam‐Tingang et al. 2020) and have also been demonstrated in vivo in a mutant mouse model (Gagnon et al. 2006). We therefore conducted further experiments on the *Clic5* mutant knock out mice to test whether the cerebellum was affected and whether balance and movement dysfunction were due to ataxia. Vestibular dysfunction with quite early onset was observed but without cerebellar involvement (Figures 2, 3, 4, 5, 6, 7). Figure 2 shows the whole cerebellum of heterozygous (*n* = 5) and homozygous (*n* = 3) *Clic5* mutant mice with no gross anatomical abnormalities; Figure 3 shows the corresponding whole cerebellar area and length (rostral–caudal and medial–lateral) measurements. Figure 4 shows histological sagittal sections of whole brain, including cerebellum; Figure 5 shows the corresponding whole brain and cerebellar areas measured in sagittal sections, in which the percent of cerebellar‐to‐whole brain area remained unchanged. Higher‐magnification histological examination showed that cerebellar foliation pattern and cerebellar lamination were not different between *Clic5* ^*−/−*^ and *Clic5* ^*+/−*^ mice (Figures 6 and 7). These findings suggest that patterning and outgrowth of cerebellar lobules are not affected in *Clic5* mutant mice.

## 4 Genetic Variants of *NEFM*

### 4.1 Two Compound‐Heterozygous Variants in the *NEFM* Gene Were Identified in Our Patients

**FIGURE 8:**
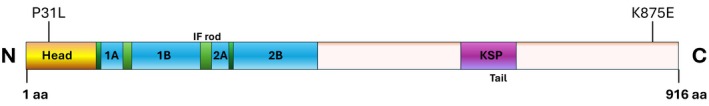
Cartoon representation of NEFM (neurofilament medium chain) protein showing the Coil 1A, 1B, 2A, and 2B regions; KSP = region with lysine–serine–proline repeats.

**FIGURE 9:**
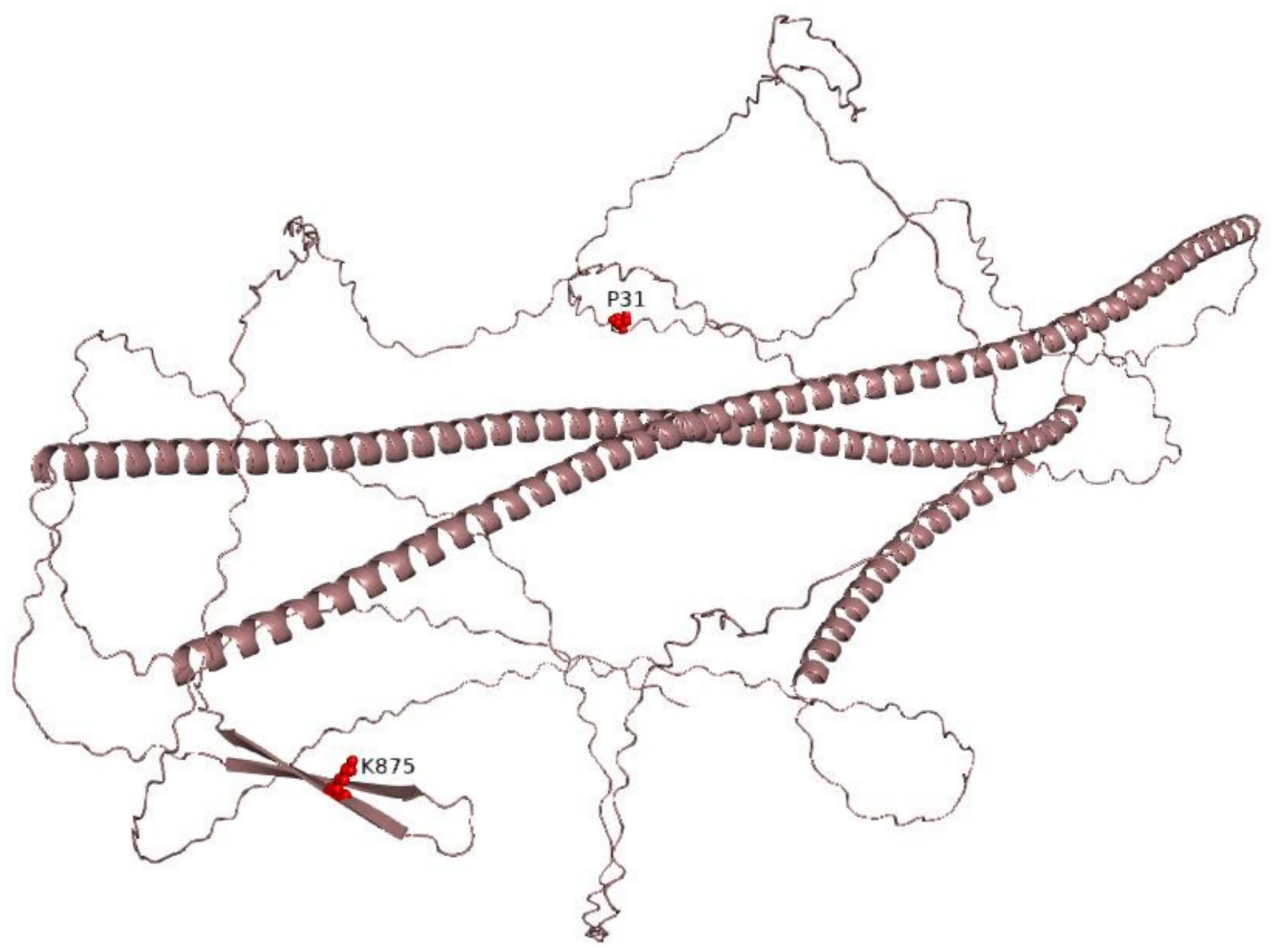
Model of *NEFM* gene structure built with PyMol (https://pymol.org/2/). Mutant residues are highlighted in red.

Missense variants Pro31Leu and Lys875Glu both have low frequencies, 3/1458368 and 5/1614122 alleles, respectively, in the gnomAD control population (Table 1). NEFM (NEuroFilament Medium chain) is a largely unfolded protein except in a neurofilament rod region (Figures 8 and 9). Variant p.(Pro31Leu) alters a phosphorylation signature of Ser30 (Lundby et al. 2012), possibly interfering with activity. The second variant p.(Lys875Glu) brings a charge inversion in the protein tail that shows compositional bias (exceedingly rich in Lys and Glu), and again has serine (Ser876) as neighbour. Scanning for kinase‐specific phosphorylation sites with GPS 5.0 (Wang et al. 2020) predicted the serines to be targeted by the CAMK kinase family and MARK with a particularly high score. The result is coherent with the significant role of the CAMK family in neuronal development (Bayer and Schulman 2019; Biernat et al. 2002; Wayman et al. 2008). NEFM variants were uploaded in GeneMatcher database (https://genematcher.org/submission/13178).

## 5 Discussion

Pure cerebellar ataxia (CA) is a subset of spinocerebellar ataxias, a heterogeneous group of neurological genetic disorders characterised by defective cerebellar development which causes incoordination and movement variability, impacting motor function, cognition and mood (Kerber et al. 2005; Marsden 2018). Comprehensive clinical examination and advanced diagnostic methods, including neuroimaging and genetic testing, are essential for accurate diagnosis (Radmard et al. 2023). Diagnosis of hereditary CA has improved with advances in molecular techniques, such as NGS and WES, which have enabled rapid detection of causative variants in multiple genes. A definitive diagnosis remains elusive in many cases, highlighting the need for ongoing research (Sailer and Houlden 2012). Here we employed WES to study a highly consanguineous family with two sisters affected by pure CA, in the search for a new diagnostic biomarker. After excluding pathogenic variants in genes already known to be correlated with CA, WES revealed potentially causative variants in two genes: *CLIC5* and *NEFM*.

The *CLIC5* (Chloride Intracellular Channel 5) gene is associated with autosomal‐recessive deafness (DFNB103, OMIM #616042) and encodes a cytoskeletal‐associated protein isolated from human placental microvilli (Berryman and Bretscher 2000). Although Clic5 has the capacity to function as a chloride channel in vitro, there is no evidence to support its function as an ion channel in vivo (Berryman et al. 2004; Gagnon et al. 2006). The *Clic5* ^*Jbg2J*^ mutant mouse (C57BL/6J‐Clic5jbg‐2 J/KjnJ; this article), which essentially mimics the inner ear phenotype of the original *Clic5* ^*Jbg*^ knockout model (Gagnon et al. 2006), was evaluated to check for possible cerebellar involvement (Berryman et al. 2004; Gagnon et al. 2006). Indeed, apart from hearing problems, the *Jbg2J* model manifests balance and movement issues. Our experiments therefore addressed whether the symptoms could also be due to cerebellar degeneration. The results of this study, especially those focused on histological examination and measurement of cerebellar area of *Jbg2J* mutant mice, ruled out a direct link between *CLIC5* and ataxia in mice. In seeking other similarities with the mouse model, our patients were examined for hearing impairment: none were found, further suggesting that the homozygous Ala114Glu missense variant in *CLIC5* is not involved in their pathology.

Next, we focused on *NEFM*, a gene that encodes the middle chain of neurofilaments (NFs), forming the cytoskeleton of myelinated fibres. Neurofilaments play essential functions throughout the nervous system, comprising axonal transport, synaptic activity, and cerebellar development (Kirkcaldie and Dwyer 2017; Riederer et al. 1996; Trojanowski et al. 1986; Yan et al. 2007; Yuan and Nixon 2016). The activity of NFs is modulated by post‐translational modifications such as phosphorylation, which play a major role in regulating their interactions (Dale and Garcia 2012; Didonna and Opal 2019). Dysregulated phosphorylation can lead to formation of toxic NF aggregates, resulting in neuronal degeneration. While NF aggregates have also been identified in the blood of healthy subjects (Adiutori et al. 2018), NF aggregation is indeed correlated with neurodegeneration and neuronal cell death (M. K. Lee et al. 1994). Plasma NFs have been proposed as biomarkers of CA (Coarelli et al. 2021; Wilke et al. 2018). Apart from pathological conditions, reduced NF phosphorylation and subsequent aggregation are also typical of ageing (Vega et al. 1994; Vickers et al. 1994). Indeed, in vivo experiments on rats demonstrated that the quantity of phosphorylated NFs is sharply reduced in old rats (Vega et al. 1994), while another in vivo experiment on transgenic mice showed that accumulation of NFs is age dependent (Vickers et al. 1994). Irrespective of NFs, protein phosphorylation and accumulation of protein aggregates are typical features of ageing. Alterations in protein kinase pathways during senescence have been shown to affect neurotransmitter function and synapses (Magnoni et al. 1991), while the aged brain contains large amounts of protein aggregates that cause progressive cell damage and organ dysfunction (Cuanalo‐Contreras et al. 2022). The importance of NEFM phosphorylation is also supported by studies on the neurotoxic effects of molecules such as diphenyl ditelluride. The neurotoxic effect of this molecule is driven by changes in the phosphorylation of NFs, affecting the cytoskeleton of striatal and cerebellar neurons (Heimfarth et al. 2012). Interestingly, NFs have also been shown to be major targets in autoimmune CA (Basal et al. 2018). The clinical picture of pure hereditary CA overlaps significantly with autoimmune CA, emphasizing that similar pathologies can result from genetic and autoimmune factors, probably involving similar molecular pathways. Transgenic mice models also support the involvement of NEFM phosphorylation and aggregation in neurodegenerative processes. Transgenic mice, expressing human NEFM only in neurons (V. M. Lee et al. 1992), develop progressive limb paralysis and NF aggregates (Gama Sosa et al. 2003; Tu et al. 1995), also with changes in the phosphorylation status of NFs (Tu et al. 1995). Finally, accumulation of NFs with dysregulated phosphorylation was also reported in a case of neurodegenerative disease with neurogenic atrophy and progressive wasting of skeletal muscles, suggesting the importance of NF phosphorylation in human pathophysiology (Wiley et al. 1987).

No disease‐causing variants in the *NEFM* gene have yet been reported in the literature (see OMIM *162250). The variants in *NEFM* identified in this study do not seem to contribute individually to CA when they occur in a single allele. However, their association with the disease becomes apparent when they occur in compound heterozygosity. Both mutant residues have important roles in NEFM function, and the genetic variants may be detrimental to NEFM activity. Indeed, p.(Pro31Leu) and p.(Lys875Glu) alter the phosphorylation signatures of Ser30 and Ser876, respectively, possibly interfering with the interaction of NEFM with CAMK family proteins (Bayer and Schulman 2019; Biernat et al. 2002; Lundby et al. 2012; Wayman et al. 2008). CAMK family proteins have a significant role in neuronal development, driving memory and neuronal plasticity, and are involved in cerebellar development (Wayman et al. 2008). Their involvement in neurodegenerative diseases has also been proposed (Sałaciak et al. 2021). CAMKII is a major player in NEFM phosphorylation, playing a significant role in NEFM aggregation. Thus, altering the phosphorylation of NEFM, as by disrupting the interaction of NEFM with CAMK family proteins and causing its aggregation, could be an important factor for neurodegeneration and CA. Based on the cumulative evidence, NEFM emerged as the most compelling candidate among the single nucleotide variants for association with CA.

To our knowledge, this is the first case where genetic variants in *NEFM* and specifically its phosphorylation sites have been proposed as responsible for a human pure CA. The main limitation of this study is the lack of whole genome sequencing, which could have revealed other pathogenic variants in non‐coding regions, and the analysis of repeat expansions and copy number variants which could be resolved applying long‐read sequencing (Erdmann et al. 2023). Anyway, our results are in line with studies in transgenic mouse models, where phosphorylation changes in NEFM lead to the formation of aggregates and neurodegeneration. Our study also underlines the role of NFs and NEFM as endpoints of multiple neurodegenerative processes, at both a physiological level, in ageing, and at a pathological level, in hereditary and autoimmune CA. The study may shed new light on the biological basis of pure hereditary CA, proposing dysregulated phosphorylation of NEFM as an important mechanism. These findings may be useful for developing targeted therapeutic approaches and for advancing the genetic diagnosis of hereditary CA, possibly enabling the screening of healthy carriers.

## 6 Conclusions

Accurate diagnosis of familial CA is challenging due to limited understanding of the genetic foundations of CA. Genetic variants of known CA‐related genes were excluded by WES in a highly consanguineous family with pure CA. A homozygous genetic variant of *CLIC5* was identified, but in vivo studies using a *Clic5* KO mouse model did not support its association with CA onset. Two compound‐sheterozygous genetic variants of *NEFM* subsequently emerged as potential contributors to CA onset. Literature reviews and *in silico* studies suggested that *NEFM* and its phosphorylation state followed by aggregate formation could be involved in neurodegeneration and CA. This article proposes *NEFM* as a possible new candidate gene for hereditary CA, and supports the involvement of NEFM phosphorylation in physiological conditions, such as ageing, and pathological conditions, such as neurodegeneration and CA.

## Author Contributions

**Matteo Bertelli:** conceptualization. **Paolo Enrico Maltese, Gabriele Bonetti, Elena Manara:** data curation. **Benedetta Tanzi:** validation. **Paolo Enrico Maltese, Elena Manara, Benedetta Tanzi:** formal analysis. **Matteo Bertelli:** funding acquisition. **Andrea Bernini, Mark A. Berryman, Soichi Tanda, Corinne Nielsen, Silvia Casagrande, Amanda Ferrero, Salvatore Stano, Riccardo Zuccarino, Andrea Barp, Pietro Chiurazzi:** investigation. **Paolo Enrico Maltese, Benedetta Tanzi:** methodology. **Paolo Enrico Maltese, Matteo Bertelli:** project administration. **Andrea Bernini:** resources/software. **Matteo Bertelli:** supervision. **Gabriele Bonetti:** visualization. **Paolo Enrico Maltese, Gabriele Bonetti:** writing – original draft. **Elena Manara, Andrea Bernini, Mark A. Berryman, Soichi Tanda, Corinne Nielsen, Silvia Casagrande, Amanda Ferrero, Salvatore Stano, Riccardo Zuccarino, Andrea Barp, Pietro Chiurazzi:** writing – review and editing. All authors have read and agreed to the published version of the manuscript.

## Funding

This research was funded by the Provincia Autonoma di Bolzano in the framework of LP 14/2006.

## Consent

All patients received pre‐test genetic counselling and provided written informed consent in compliance with the Declaration of Helsinki.

## Conflicts of Interest

All affiliations of the authors with private companies have been declared to make clear the position regarding the interests of these companies. The authors are affiliated with private companies for which there could be a possible conflicts of interest. The authors of this article are reported to be patent inventors.

## Supporting information


**Table S1:** List of homozygous genetic variants.

## Data Availability

The data that support the findings of this study are available on request from the corresponding author. The data are not publicly available due to privacy or ethical restrictions.
