# Supplementary material for: Exclusion of CLIC5 as a Candidate Gene and Identification of NEFM as a Possible Novel Gene Correlated With Autosomal Recessive Pure Cerebellar Ataxia in a Highly Consanguineous Family
Source: Mol Genet Genomic Med. 2026 Mar 30;14(4):e70199. doi: 10.1002/mgg3.70199 (PMC13140635; doi:10.1002/mgg3.70199)
Supplement: Supplementary file 1 — Table S1: List of homozygous genetic variants. [file MGG3-14-e70199-s001.docx]

SUPPLEMENTARY MATERIALS

| **ID** | **GENE** | **MAF** | **Nucleotide change** | **Amino acid change** |
| --- | --- | --- | --- | --- |
| rs62621173 | IFI16 | 0.03928 | NM_001206567.1:c.1535C>T | NP_001193496.1:p.Ser512Phe |
| rs62061174 | NLGN2 | 0.02359 | NM_020795.3:c.2264C>T | NP_065846.1:p.Ala755Val |
| rs151174418 | DNAH9 | 0.002332 | NM_001372.3:c.2519A>G | NP_001363.2:p.Asp840Gly |
| rs9472017 | PTK7 | 0.01836 | NM_001270398.1:c.2259G>C | NP_001257327.1:p.Glu753Asp |
| rs143360018 | CLIC5 | 0.002836 | NM_001114086.1:c.818C>A | NP_001107558.1:p.Ala273Glu |
| rs192244210 | COL21A1 | 0.01382 | NM_001318751.1:c.1748C>G | NP_001305680.1:p.Pro583Arg |
| rs35629782 | MB21D1 | 0.05102 | NM_138441.2:c.143C>A | NP_612450.2:p.Ala48Glu |
| rs1830080 | ZNF273 | 0.04386 | NM_021148.2:c.1361G>A | NP_066971.2:p.Gly454Glu |

Table S1. List of homozygous genetic variants.
